# New insights into *Saccharomyces cerevisiae* induced calcium carbonate precipitation

**DOI:** 10.3389/fbioe.2023.1261205

**Published:** 2023-08-31

**Authors:** Tianxiao Li, Huabing Zhang, Xiang Tan, Rui Zhang, Fasi Wu, Zongren Yu, Bomin Su

**Affiliations:** ^1^ Dunhuang Academy, The Conservation Institute, Dunhuang, China; ^2^ National Research Center for Conservation of Ancient Wall Paintings and Earthen Sites, Dunhuang, China; ^3^ Joint International Research Laboratory of Environmental and Social Archaeology, Shandong University, Qingdao, China; ^4^ Institute of Cultural Heritage, Shandong University, Qingdao, China

**Keywords:** *Saccharomyces cerevisiae*, microbially induced calcium carbonate precipitation, TCA cycle, initial pH, organic acids

## Abstract

Our previous study reported that *Saccharomyces cerevisiae* could induce calcium carbonate (CaCO_3_) precipitation, but the associated mechanism was unclear. In the present study, *Saccharomyces cerevisiae* was cultured under various conditions, including the presence of different organic acids and initial pH, and the yields of CaCO_3_ formation induced by the different organic acids were compared. The metabolism of organic acid by the metabolites of *S. cerevisiae* was also assessed *in vitro*. The SEM-EDS and XRD results showed that only acetate acid, pyruvic acid, and α-ketoglutaric acid could induce CaCO_3_ formation, and the weight order of the produced CaCO_3_ was pyruvic acid, acetate acid, α-ketoglutaric acid. In addition, the presence of only yeast metabolites and the initial neutral or alkaline environment also limited the CaCO_3_ formation. These results illustrated that organic acid oxidation intracellularly, especially the tricarboxylic acid cycle, was the major mechanism, and the CaCO_3_ yield was related to the amount of CO_2_ produced by the metabolism of organic acids. These findings will deepen the knowledge of the mineralization capacity of *S. cerevisiae* and provide a theoretical basis for the future application of yeast as an alternative microorganism in MICP.

## Introduction

Microbially induced calcium carbonate precipitation (MICP) is a common phenomenon in nature, and the formed calcium carbonate (CaCO_3_) has become a new green material used in numerous applications, including soil amelioration, building material rehabilitation, and the conservation of stone monuments (Dhami et al., 2013; 2014; Lin et al., 2021; Ortega-Morales and Gaylarde, 2021; Ortega-Villamagua, E., Gudiño-Gomezjurado, M. and Palma-Cando, 2020; Reeksting et al., 2020; Zhang et al., 2023). Urea hydrolysis is the major pathway for the application of MICP because of the higher and faster precipitation rate of CaCO_3_ (Reeksting et al., 2020; Justo-Reinoso et al., 2021; Lin et al., 2021). However, the associated byproducts, such as ammonia and nitrogen oxide, are environmentally toxic and have a negative impact on the substrate (Reeksting et al., 2020; Justo-Reinoso et al., 2021; Lin et al., 2021; Sidhu et al., 2022). To reduce the environmental impact of ureolytic strains, microorganisms with other pathways of CaCO_3_ production are also being tested in MICP applications. Oxidation of organic acids is also a pathway of MICP, and the final products are carbon dioxide and water, which are not harmful to the environment (Dhami et al., 2014). In addition, the precipitation of CaCO_3_ induced by the oxidation of organic acids takes place at a slower rate in comparison with the hydrolysis of urea, increasing the depth and efficacy of the restoration (Reeksting et al., 2020; Justo-Reinoso et al., 2021). Our previous study showed that *Saccharomyces cerevisiae* could induce CaCO_3_ precipitation through the oxidation of organic acids (Li and Li, 2022). This suggests that *Saccharomyces cerevisiae* can be an alternative microorganism for MICP applications.

As shown in Equations 1-3 (Dhami et al., 2014), when organic acids are the main carbon and energy source, their consumption will produce CO_2_ and increase the surrounding pH, and the presence of Ca^2+^ favors calcium precipitation as CaCO_3_. This process can occur both intracellularly and extracellularly and is influenced by several factors. For intracellular process, the type of organic acids and nutrients, pH and some metal ions can influence CaCO_3_ formation by affecting cell growth, organic acid uptake and inorganic carbon production (Wolf et al., 2003; Peña et al., 2015; Li and Li, 2022). In the extracellular process, proteins secreted by microorganisms consume the organic acids extracellularly to raise the pH and produce inorganic carbon for CaCO_3_ formation (Yafeng and Chunxiang, 2022). However, some organic matters from metabolites can inhibit the CaCO_3_ precipitation by competitive adsorption of calcium ions (Rui et al., 2021; Robles-Fernández et al., 2022). Our previous study has confirmed that the type of organic acids can influence the process of calcium carbonate precipitation induced by *Saccharomyces cerevisiae*, but the further mechanism of this process is not yet clear.

$${CH}_{3}{COO}^{-}+2O_{2}\;\to \;2{CO}_{2}+H_{2}O+{OH}^{-}$$ (1)

$$2{CO}_{2}+{OH}^{-}\;\to \;{CO}_{2}+{HCO}_{3}^{-}$$ (2)

$$2{HCO}_{3}^{-}+{Ca}^{2+}\;\to \;{CaCO}_{3}+{CO}_{2}+H_{2}O$$ (3)

In the present study, we explored the factors influencing yeast-induced CaCO_3_ precipitation by adjusting different types of organic acid and the initial pH of the medium. In addition, yeast metabolites were used to degrade acetic acid *in vitro* to determine whether the metabolic process of organic acids occurs intracellularly or extracellularly. Finally, we compared the yield of CaCO_3_ produced by yeast under different factors. This study further elucidated the mechanism of yeast-induced CaCO_3_ precipitation and provided a theoretical basis for the application of yeast in MICP.

## Materials and methods

### Cultivation of *Saccharomyces cerevisiae* under various conditions

Instant active dry yeast, which is identified as *S. cerevisiae*, was purchased from Angel Yeast Co., Ltd (China). This strain is a common commercial product on the market, which has the advantages of easy availability and low cost, and it was activated and purified with B4 medium (3.52 g/L calcium acetate monohydrate, 4 g/L yeast extract, 10 g/L glucose). Seven organic acids or their calcium salts were used to culture yeast. The detailed composition of the modified B4 media is presented in Table 1 (a–g). The concentrations of Ca^2+^ and yeast in each media were 0.67 g/L and 1 × 10^5^ CFU/mL, respectively, and the initial pH was adjusted to 6. In addition, two media, with initial pH of 7 (Table 1) h and 8 (Table 1) i, were also used to culture yeast.

**TABLE 1:** The composition of media under different conditions.

Group	Yeast extract	Calcium source	Other additives
a	+	calcium acetate	-
b	+	calcium pyruvate	-
c	+	Ca (OH)_2_	succinic acid
d	+	Ca (OH)_2_	α-ketoglutaric acid
e	+	Ca (OH)_2_	malic acid
f	+	Ca (OH)_2_	formic acid
g	+	Ca (OH)_2_	propanoic acid
h^a^	+	calcium acetate	NaOH
i^b^	+	calcium acetate	NaOH

^a^ , pH = 7.

^b^ , pH = 8.

Three parallel experiments were performed for each medium, and yeast was incubated at 30 C for 7 days. The final pH of the media was measured using an S210-K pH meter (Mettler Toledo, Switzerland). The biomasses were filtered with filter paper, washed with deionized water and absolute alcohol, and dried at 60 C to constant weight.

### Comparison of CaCO_3_ yields from cultures with different organic acids

Three acids, acetate acid, pyruvic acid, and α-ketoglutaric acid, related to CaCO_3_ formation, were selected to culture yeast, and the biomass weight was used as an indicator of the yield of CaCO_3_ (Li and Li, 2022). Yeast was incubated as described 2.1, and the dry weight of the biomass and the final pH of the media were recorded.

### The role of *S. cerevisiae* metabolites in organic acid oxidation

Yeast was cultured with the media (Table 1) a for 2 days, and then the media were filtered through 0.22 μm membranes. Afterward, 10 mL of a sterile solution of calcium acetate or calcium chloride (the concentration of Ca^2+^ was 0.2 mol/L) was mixed with the medium without yeast. These solutions continued to be cultured at the same condition for 5 days. Centrifugation (5,000 rpm and 10 min) was then carried out and the deposition that might be present were washed with deionized water and absolute alcohol, and dried at 60 °C to constant weight.

### Characterization of *S. cerevisiae*-induced crystals

The biomasses obtained from the above experiments were analyzed with scanning electron microscope and energy-dispersive X-ray spectroscopy (SEM-EDX), X-ray diffraction (XRD) and Fourier-transform infrared spectroscopy (FTIR). After grinding, the morphological characteristics were observed via SEM (Quattro S, Thermo Fisher, United States), and the elemental composition of the minerals was analyzed via EDX (XFlash 6,160, Bruker, Germany). Both SEM and EDX were conducted under an accelerating voltage of 15 kV. The crystal of the formed minerals was characterized via XRD (D8 Advance, Bruker, Germany), and the samples were analyzed over the 2θ range of 10°–70° at a scan rate of 1°/min in 0.02° increments. Additionally, the biomass was analyzed via FTIR (Nicolet iN 10, Thermo Fisher, United States) with potassium bromide pellets to determine whether it contained the unmetabolized organic calcium.

## Results and discussion

### Factors influencing *S. cerevisiae*-induced CaCO_3_ precipitation

**FIGURE 1:**
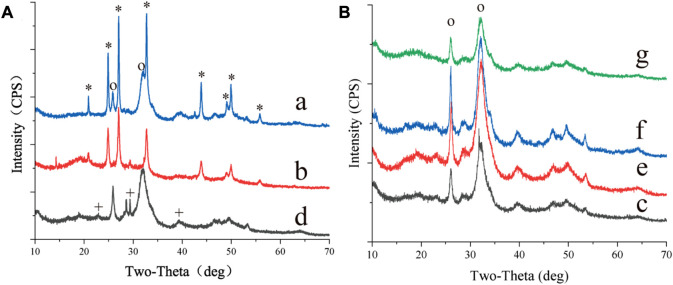
XRD of minerals synthesized by *S.cerevisiae* with different culturing conditions. **(A)** showed the results of the organic acids **(a)** acetate **(b)** pyruvate and **(d)** α-ketopentate in the medium, **(B)** showed the results of the organic acids **(c)** succinate **(e)** malate **(f)** formate **(g)** propionate in the medium. *, the base peak of vaterite, +, the base peak of calcite, o, the base peak of calcium phosphate.

**FIGURE 2:**
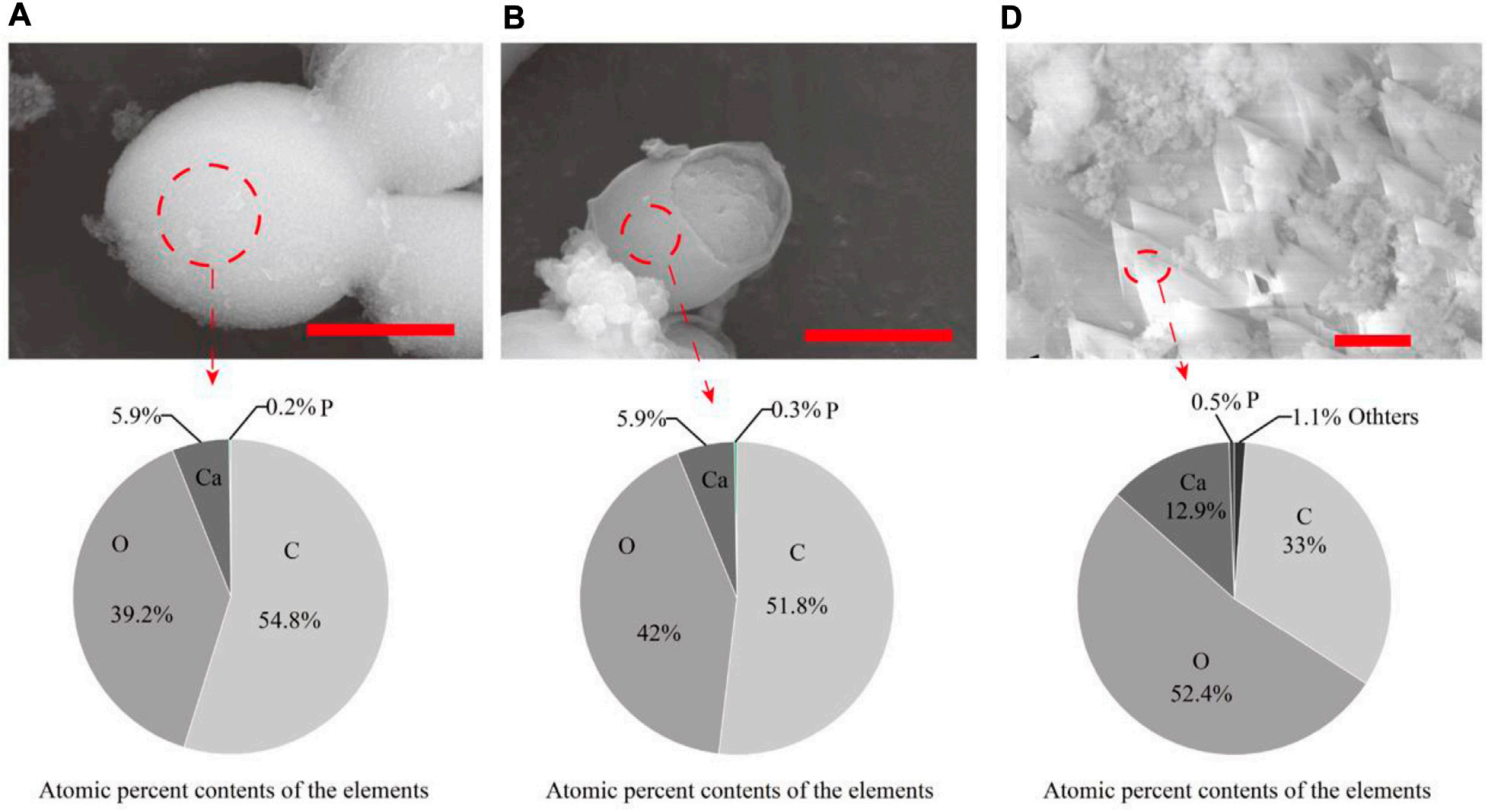
SEM-EDS of CaCO_3_ synthesized by *S*.*cerevisiae* with different culturing conditions. **(A)**acetate **(B)** pyruvate **(D)** α-ketopentate in the media. The red bar was 2 μm. The atomic percent contents refer to the elemental composition and proportion of minerals contained in the circled area.

*S. cerevisiae* was cultured using seven organic acids, and minerals were widely present around the yeast cells cultured with all organic acids (Supplementary Figure S1). Most of the minerals were identified as calcium phosphate (Supplementary Figure S1, Figure 1B). The findings demonstrated that yeast had a high phosphatase activity and could secrete a large amount of phosphate outside the cell (Krumov et al., 2009; Krumov and Posten, 2011; Durán and Favaro, 2018; Qin et al., 2020; Geetha et al., 2021). CaCO_3_ was only found in the biomass cultured with acetate, pyruvate, and α-ketoglutarate (Figures 1A, 2). In addition, the pH results showed that the metabolism of acetate, pyruvate, α-ketoglutarate, and propionate increased the media from acidic to alkaline. Particularly, the pH of the media containing acetate or pyruvate was above 8. The alkaline environment and amount of dissolved inorganic carbon (DIC) are key factors affecting the process of CaCO_3_ formation by microorganisms (Li et al., 2018). It indicated that the consumption of acetate, pyruvate and α-ketoglutarate by yeast also provided sufficient CO_2_, the main source of DIC according to Equations 1–3, for CaCO_3_ formation. However, no CaCO_3_ was found in the biomass cultured with propionate, similar to our previous study with lactate (Li and Li, 2022). The failure of the metabolism of these organic acids to produce sufficient CO_2_ may be the main reason. The above results suggested that the type of organic acids was one of the key factors influencing yeast-induced CaCO_3_ mineralization.

*S. cerevisiae* was also cultured at different initial pH values, including 6, 7 and 8. However, the pH of the initial neutral and alkaline media decreased to 6.76 and 7.59, respectively, after 7 days of incubation, and no CaCO_3_ was found in the biomasses (Supplementary Figure S2). Peña et al. (2015) reported that the yeast growth rate remarkably decreased at pH 8.0. High pH also inhibits the uptake of acetate by yeast, but would not affect its metabolism (Mills, 1972; Pera et al., 1972; Peña et al., 2015). In addition, yeast has a high acidification capacity (Conway and Downey, 1950; Peña et al., 2015), and the medium was mainly acidified by CO_2_ produced by glucose metabolism (Peña et al., 2015). In the present study, neutral and alkaline environments inhibited yeast growth rate and acetate uptake, resulting in a reduction of OH^−^ from acetate metabolism. In contrast, CO_2_ and organic acids produced by metabolic activities resulted in a small decrease in the pH of the medium. CO_2_ was mainly in the form of bicarbonate because the pH was less than 8.0, resulting in the nonexistence of CaCO3 in the biomasses. These suggested that acidic initial environment was one of the necessary conditions for CaCO_3_ precipitation induced by yeast.

Overall, yeast-induced CaCO_3_ precipitation was influenced by several factors. First, the presence of yeast extract is necessary for carbonate precipitation (Li and Li, 2022). Second, the initial media should be acidic. Lastly, organic acid is also required. When organic acid was used as the main carbon source, only acetate, pyruvate, and α-ketoglutarate contributed to CaCO_3_ biomineralization, and when glucose was used as the substrate, other organic acids such as lactate and pyruvate also contributed to the process.

Compared to other ureolytic and non-ureolytic microorganisms (Ortega-Villamagua, E., Gudiño-Gomezjurado, M. and Palma-Cando, 2020; Justo-Reinoso et al., 2021; Reeksting et al., 2020), the conditions for yeast-induced calcium carbonate deposition are somewhat limited, which consequently limits its potential application within MICP. However, yeast also has advantages in inducing CaCO_3_ precipitation. As one of the most widely used microorganisms in brewing and the production of food (Ma et al., 2011), yeast is inexpensive and readily available from the market. In addition, as a widely used model organism, gene modification techniques are well developed in yeast. For example, Barbero et al. (2013) have successfully edited the genes encoding carbonic anhydrase and mineralization peptides into yeast, significantly enhancing the ability to induce CaCO_3_ precipitation. From these perspectives, yeast can be considered as a potential candidate microorganism for MICP applications.

### Metabolic pathway of *S. cerevisiae*-related MICP

The metabolites of yeast after 2-day cultivation were incubated with calcium acetate and calcium chloride, respectively for 5 days, but no precipitation occurred. This indicated that the consumption of organic acids by yeast only occurred intracellularly.

Transcriptome analysis in our previous study showed that the addition of organic acids could upregulate the genes in many pathways, including the tricarboxylic acid (TCA) cycle (Li and Li, 2022). All three acids capable of inducing calcium carbonate precipitation are associated with the TCA cycle. Acetic acid and pyruvic acid can be transferred to acetyl-CoA, the substrate of the TCA cycle. α-ketoglutaric acid is also an intermediate product in the TCA cycle. According to the pathway, one molecule of pyruvic acid generates three molecules of CO_2_, while one molecule of acetic acid produces two molecules of CO_2_, and one molecule of α-ketoglutaric acid only generates one molecules of CO_2_. We compared the CaCO_3_ production induced by the three organic acids. The yields of CaCO_3_ produced using the three organic acids increased in the following order: α-ketoglutarate < acetate < pyruvate (Supplementary Figure S3), similar to the case of CO_2_ yields.

Citric acid metabolism based on the TCA cycle can also produce CO_2_, but the low solubility of calcium citrate allows only a few citrates to enter the yeast cells. Our previous study showed that a large amount of calcium citrate occurred in the biomass after cultivation (Li and Li, 2022). The low utilization rate of citrate could not increase the pH of the medium and produce sufficient CO_2_ for CaCO_3_ precipitation. Succinic acid and malic acid are also intermediate products in the TCA cycle, and their calcium salts could be absorbed by yeast (Barnett and Kornberg, 1960). Fourier-transform infrared spectroscopy results showed that no calcium succinate and calcium malate occurred in the final biomass (results not shown). However, these acids neither increased the pH nor promoted CaCO_3_ formation. In the TCA cycle, the metabolism of these acids did not produce CO_2_. However, they can also produce CO_2_ via the glycolytic/glycogenic pathway, but this process does not involve the degradation of organic acids and does not produce OH^−^. These factors contributed to the inability of succinic acid and malic acid to contribute to yeast-induced CaCO_3_ precipitation.

**FIGURE 3:**
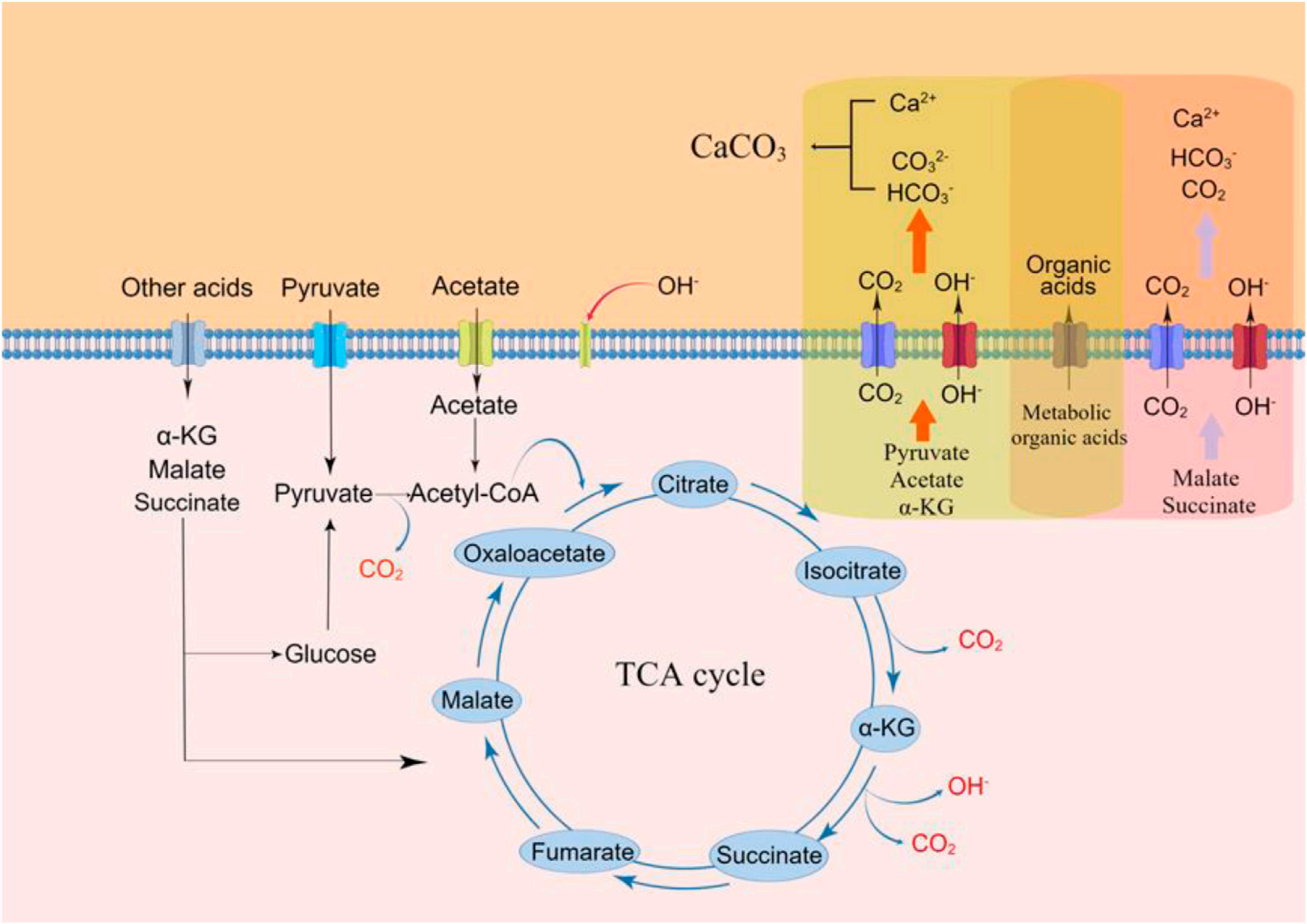
Mechanism of *S*.*cerevisiae* induced calcium carbonate precipitation.

The above results suggest that the TCA cycle is the main pathway related to yeast-induced CaCO_3_ precipitation and that organic acids oxidized to produce CO_2_ and OH^−^ in this pathway are involved in the process (Figure 3, drawn by Figdraw).

## Conclusion

The study suggested that organic acids such as acetic acid, pyruvic acid and α-ketoglutarate, as well as the initial acidic pH were the factors driving CaCO_3_ precipitation by yeast. The TCA cycle was the main pathway for yeast-included CaCO_3_ precipitation. Oxidation of organic acids was occurred intracellularly and the amount of CO_2_ produced in the process was related to the amount of calcium carbonate precipitated. This study further explored the mechanisms of *S. cerevisiae* in MICP and suggested that *S. cerevisiae* could be an alternative microorganism for MICP applications.

## Data Availability

The original contributions presented in the study are included in the article/Supplementary Material, further inquiries can be directed to the corresponding authors.
