# Supplementary material for: New insights into Saccharomyces cerevisiae induced calcium carbonate precipitation
Source: Front Bioeng Biotechnol. 2023 Aug 31;11:1261205. doi: 10.3389/fbioe.2023.1261205 (PMC10500597; doi:10.3389/fbioe.2023.1261205)
Supplement: Supplementary file 1 [file DataSheet1.docx]

**New insights into *Saccharomyces cerevisiae* induced calcium carbonate precipitation**

Tianxiao Li^1,2,3,4^, Huabing Zhang^1,2^, Xiang Tan^1,2^, Rui Zhang^1,2^, Fasi Wu^1,2^, Zongren Yu^1,2^, Bomin Su^1,2*^

^1^Dunhuang Academy, The Conservation Institute, Dunhuang 736200, Gansu, China

^2^National Research Center for Conservation of Ancient Wall Paintings and Earthen Sites, Dunhuang 736200, Gansu, China

^3^Joint International Research Laboratory of Environmental and Social Archaeology, Shandong University, Qingdao, Shandong, 266237, China

^4^Institute of Cultural Heritage, Shandong University, Qingdao, Shandong, 266237, China

*Corresponding Author: Bomin Su, Email: suboming@hotmail.com

**Supplement Contents**

This supplement contains, in the following order:

Figure S1. SEM-EDS of calcium phosphate synthesized by *S.cerevisiae* with different culturing conditions.

Figure S2. XRD of minerals synthesized by *S.cerevisiae* with different culturing conditions.

Figure S3. The weight of CaCO_3_ induced by *S.cerevisiae*. (a)acetate (b) pyruvate (d) α-ketopentate in the media.


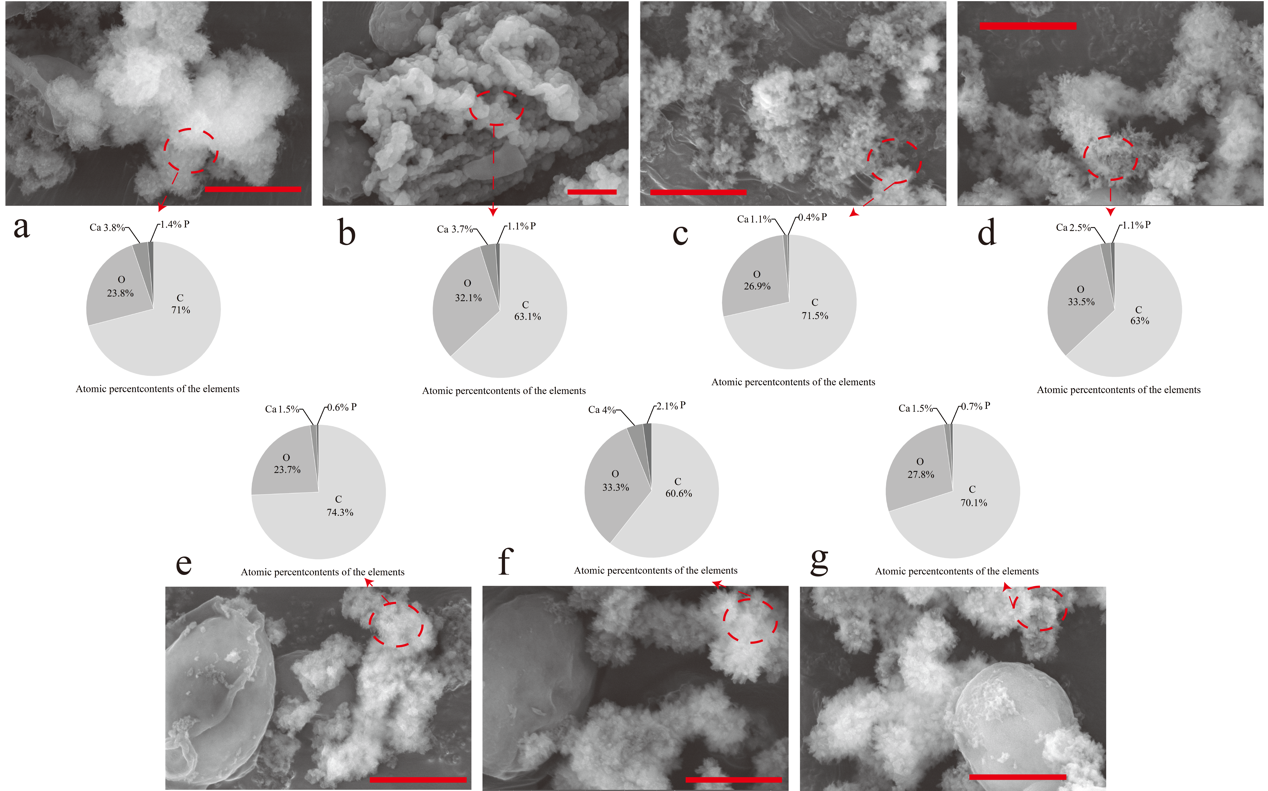


Figure S1 SEM-EDS of calcium phosphate synthesized by *S.cerevisiae* with different culturing conditions. (a)acetate (b) pyruvate (c) succinate (d) α-ketopentate (e) malate (f) formate (g) propionate in the medium. The red bar was 2 μm.


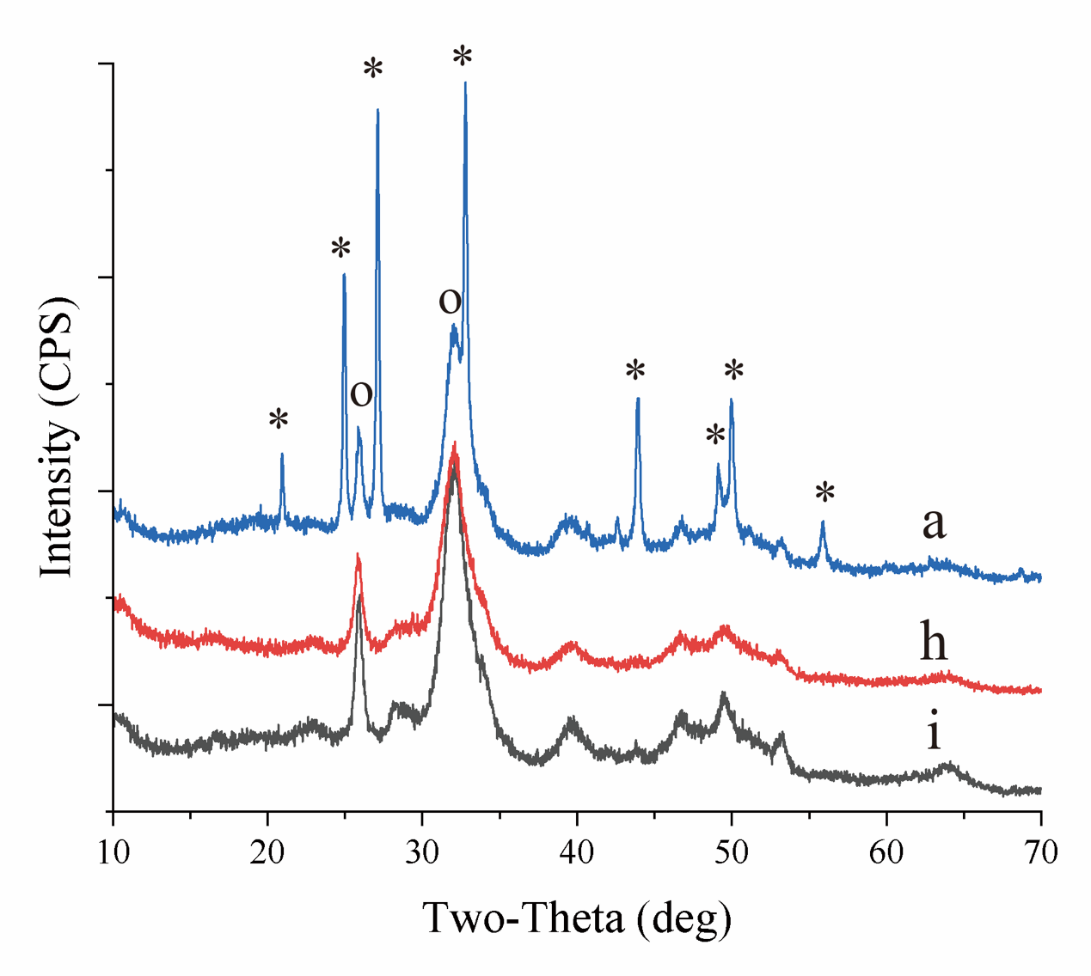


Figure S2 XRD of minerals synthesized by *S.cerevisiae* with different culturing conditions. (a)pH=6, (h) pH=7 (i) pH=8. *, the base peak of vaterite, o, the base peak of calcium phosphate.





Figure S3 The weight of CaCO_3_ induced by *S.cerevisiae*. (a)acetate (b) pyruvate (d) α-ketopentate in the media. All the groups were significantly different with each other (*p*<0.05)
